# Cloning and transcriptional analysis of the mouse receptor activity modifying protein-1 gene promoter

**DOI:** 10.1186/1471-2199-6-7

**Published:** 2005-03-24

**Authors:** Marc D Pondel, Richard Mould

**Affiliations:** 1 St. George's Hospital Medical School, Department of Cellular and Molecular Medicine, Cranmer Terrace, London, SW17 ORE, UK

## Abstract

**Background:**

Receptor activity modifying protein-1 (RAMP-1) is a single transmembrane-domain protein required for the functional expression of calcitonin gene-related peptide (CGRP) receptors. To date, little is known about the molecular mechanism(s) that activate/inhibit RAMP-1 gene expression. Such mechanism(s) are likely to play a major role in modulating the responsiveness of tissues to CGRP.

**Results:**

To initiate studies on the transcriptional regulation of the mouse RAMP-1 gene, RAMP-1 transcriptional initiation sites were mapped in a variety of tissues. Analysis of RAMP-1 expression in C2C12 myoblasts demonstrated that RAMP-1 mRNA is expressed at greatest levels in confluent myoblasts verses non-confluent and fused myoblasts. Transfection of confluent C2C12 myoblasts and NIH 3T3 cells with RAMP-1 promoter/luciferase deletion constructs revealed that 4.7 kb of RAMP-1 5' flanking region demonstrated optimal promoter activity while 343 bp of 5' flanking region was defined as a minimal RAMP-1 promoter. In non-RAMP-1 expressing HEK293 cells, constructs containing 4.7 kb to 782 bp of RAMP-1 5' flanking region were transcriptionally inactive. However, deletion of sequences -782 to -343 activated RAMP-1 promoter activity.

**Conclusion:**

These findings suggest that tissue specificity of RAMP-1 gene expression is mediated by a negative acting transcription factor that represses RAMP-1 gene expression in non-RAMP-1 expressing tissues. This transcription factor is therefore likely to play an important role in modulating the responsiveness of tissues to CGRP.

## Background

The calcitonin gene-related peptide (CGRP) belongs to a family of related peptides that includes calcitonin (CT), adrenomedullin (AM) and amylin (AMY) [1,2]. To date, CGRP is one of the most potent endogenous vasodilatory peptides discovered. CGRP mediates sensory neurotransmission and inhibits insulin action on carbohydrate metabolism [2]. CGRP has been shown to modulate immune function by inhibiting the proliferation of T cells and synthesis of T cell-derived cytokines IL-2 and IFN-γ [3-6]. In the lung, CGRP mediates multiple effects some of which have potential implications in airway homeostasis [7]. CGRP has also been shown to have cardioprotective effects in rats and humans [8,9]. In skeletal muscle, CGRP potentiates muscle contraction [10], increases the numbers of acetylcholine receptors (AchR) [11-13] and their rate of desensitisation [14]. In addition, CGRP locally increases the rate of blood flow following muscle contraction [15-17].

The effects of CGRP are mediated by CGRP receptors that are generated by a complex of proteins [18]. CGRP receptors are formed by the interaction of two separate proteins. The first protein component is the calcitonin receptor like (CL) receptor. The CL receptor is a seven transmembrane-domain receptor but is inactive when expressed in cells alone [19]. The second protein component required for CGRP receptor function is receptor activity modifying protein-1 (RAMP-1). RAMP-1 acts as a molecular chaperone and is required for the transportation of the CL receptor to the cell surface in addition to pharmacologic specificity [18].

RAMPs are a recently identified group of single transmembrane-domain accessory proteins. To date, three members of the RAMP family have been identified (RAMP-1, RAMP-2 and RAMP-3) [18]. All share 30% sequence identity, differ in their tissue distributions and are comprised of approximately 160 amino acids that make up a large extracellular N-terminal domain, a single membrane-spanning domain and a short cytoplasmic domain [18,20]. Recently, Christopoulos et al. [21] demonstrated that RAMPS interact with a number of Class II G protein-coupled receptors (GPCRs) in addition to the CL receptor. These include the vasoactive intestinal polypeptide/pituitary adenylate cyclase activating peptide receptor (VPAC1R), the glucagon and parathyroid hormone receptors (PTH1 and PTH2). VPAC1R/RAMP-2 heterodimers display a significant enhancement of agonist-mediated phosphoinositide hydrolysis compared with VPAC1R alone. This suggests that RAMPs may play a more general role in modulating cell signalling through other GPCRs than previously thought.

Despite the crucial role RAMP-1 plays in the generation of CGRP receptors, little is known about the molecular mechanism(s) regulating RAMP-1 gene expression. It is likely that such mechanism(s) play an important role in modulating the responsiveness of specific tissues to CGRP. To initiate studies on RAMP-1 gene regulation, we cloned and characterised the mouse RAMP-1 gene promoter. Analysis in three different RAMP-1 positive mouse tissues revealed multiple start sites of transcription. RT-PCR analysis of RAMP-1 mRNA in the C2C12 myoblast cell line demonstrated that endogenous RAMP-1 gene expression was greatest in confluent cultures compared to non-confluent or fused cells. Through the use of RAMP-1 promoter/luciferase constructs transfected into C2C12 myoblast cells and NIH 3T3 cells, we studied the effects RAMP-1 promoter deletions had on RAMP-1 transcriptional activity. We further demonstrated that the RAMP-1 promoter activity was tissue-specific and not expressed in the RAMP-1 negative cell line HEK293. Finally, we identified a repressor element in the RAMP-1 promoter which when deleted activates RAMP-1 promoter activity in HEK293 cells. This suggests the RAMP-1 gene is negatively regulated in non-RAMP-1 expressing cells.

## Results

### Determination of RAMP-1 transcriptional initiation sites and PCR cloning the mouse RAMP-1 promoter

To date, the transcriptional initiation sites for any mouse RAMP transcript have not yet been determined. We therefore performed 5' rapid amplification of cDNA ends (RACE) analysis to map RAMP-1 transcriptional initiation sites. For these experiments, RNA from a number of known RAMP-1 positive mouse tissues (heart, brain and skeletal muscle) was employed. Sequence analysis of 8–10 5' RACE clones from each tissue demonstrated multiple start sites of transcription (Table 1). All transcriptional start sites in mouse heart, brain and skeletal muscle occurred within a 50 bp region directly 5' to RAMP-1's start site of translation. As demonstrated by the DNA sequence analysis above, this region is highly GC rich (78%). These results suggest that RAMP-1 transcriptional initiation sites do not significantly vary between the different RAMP-1 positive tissues tested.

**Figure 1:**
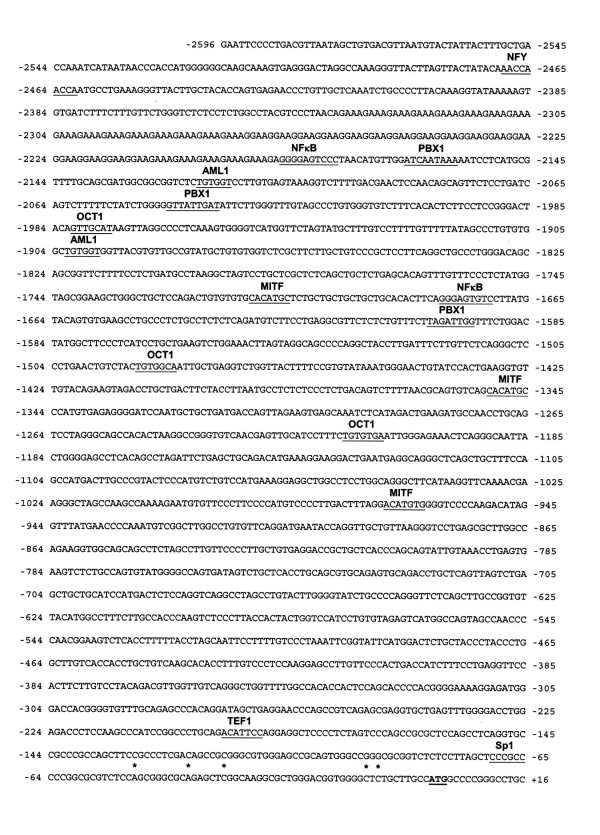
DNA sequence of mouse RAMP-1 5' flanking region. Potential transcription factor binding sites have been underlined. Underlined ATG sequence is start site of translation = 0. Transcriptional initiation sites mapped by 5' RACE analysis are indicated by *asterisks.

Having identified RAMP-1's major transcriptional initiation sites, we next wished to clone and characterise the mouse RAMP-1 gene promoter. Since extensive sequence data both 5' and 3' to RAMP-1's transcriptional start sites is available on the mouse genome data base (Accsession no. NT_039173.2) we employed RAMP-1 specific primers and PCR on mouse genomic DNA to generate a DNA fragment containing 4.7 kb of RAMP-1 5' flanking region. Promoter sequence motifs in the proximal 2.5 kb of 5' flanking region corresponding to consensus binding sites for a variety of transcription factors are present (Fig. 1). These include binding sites for the transcription factors NFκB, PBX1, AML1, OCT1, MITF, TEF1 and Sp1. Of note is a 147 bp region (-2188 to -2336) containing multiple GAAA and GGAA repeat sequences and a highly GC rich (78%) region of 144 bp region immediately 5' of the start site of translation (ATG). This GC rich region contains a potential binding site for the transcription factor Sp1.

### RAMP-1 gene expression in C2C12 myoblasts and myotubes

**Figure 2:**
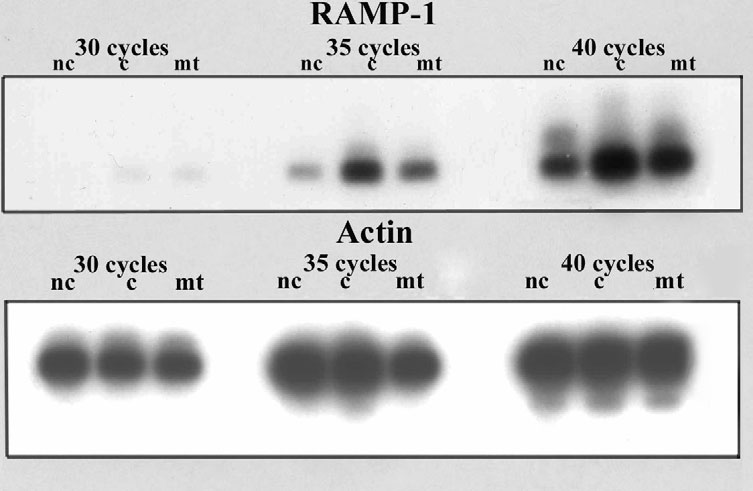
Semi-quantitative RT-PCR on RAMP-1 gene expression in C2C12 cells. Total RNA was isolated from C2C12 non-confluent cells (**nc**), confluent (**c**) and myotubes (**mt**). The RNA was subjected to reverse transcription followed by PCR with mouse RAMP-1 and actin specific primers. The PCR reactions were stopped after 30, 35, 40 cycles and size fractionated on a 1% agarose gel. The DNA was then Southern blotted overnight and hybridised with a ^32^P end-labelled RAMP-1 and actin specific probes followed by autoradiography. This experiment is representative of three experiments each giving similar results.

Northern blot analysis has demonstrated that the RAMP-1 gene is expressed in mouse skeletal muscle [20]. Since a variety of muscle cell lines are widely available and are easily transfected with DNA constructs, we decided to employ such cell lines for our studies on the transcriptional regulation of the mouse RAMP-1 gene. Before undertaking transfection experiments in a muscle cell line, we first studied endogenous RAMP-1 gene expression in the myoblast cell line C2C12. Upon incubation with reduced serum levels and high cell density, C2C12 myoblasts fuse and form myotubes. Pittner et al. [22] previously demonstrated that incubation of this cell line with CGRP induces an increase in intracellular levels of cAMP making it highly likely this cell line expresses RAMP-1 mRNA. We therefore assayed RAMP-1 gene expression in this cell line by semi-quantitative RT-PCR analysis on total RNA from non-confluent, confluent and fused C2C12 myoblasts. Southern blot analysis of RT-PCR products from these cells demonstrated the presence of RAMP-1 mRNA under all culture conditions (Fig. 2). However, RAMP-1 mRNA levels appeared to be highest in confluent versus non-confluent and fused C2C12 myoblasts. Densitomitry demonstrated that RAMP-1 mRNA expression was at least 6 times higher in confluent vs. non-confluent myoblasts.

### RAMP-1 promoter deletional analysis in transiently transfected C2C12 myoblasts

**Figure 3:**
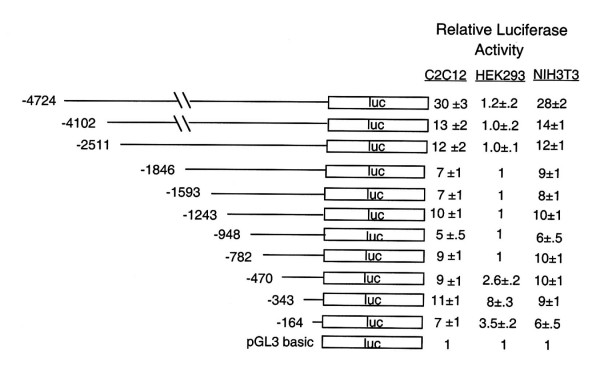
RAMP-1 promoter/luciferase activity in RAMP-1 positive and negative cell lines. RAMP-1 promoter-deletion constructs were generated by PCR and ligated into the luciferase expression vector pGL3-basic. The amount of 5' flanking region relative to the start site of translation (ATG = 0) is indicated for each construct. Constructs were transiently transfected into RAMP-1 positive expressing cells (C2C12 and NIH3T3) and RAMP-1 negative cells (HEK293). Forty-eight hours after transfection, cells were harvested and assayed for luciferase activity. The co-transfection control plasmid pRL-TK was employed for all transfections. Relative luciferase activities were determined by comparing luciferase activity of cells transfected with RAMP-1/luciferase deletion constructs to cells transfected with the promoterless pGL3-basic plasmid. Background levels of luciferase = 1. The data are an average of three independent sets of transfections each giving similar results.

To delineate potential transcriptional regulatory elements in the 4.7 kb RAMP-1 promoter, a series of 5' RAMP-1 promoter/luciferase constructs were generated. Luciferase analysis on transfected confluent C2C12 myoblasts revealed that a RAMP-1 promoter/luciferase construct with approximately 4.7 kb of 5' flanking region (relative to RAMP-1's start site of translation) had the greatest levels of luciferase activity (30 fold over the background plasmid pGL3 basic). Deletion of approximately 700 bp of 5' flanking region (to -4102) caused up to a 55% drop in expression levels. Deletions to -343 of RAMP-1 5' region did not cause additional decreases in promoter activity. However, deletion to -164 reduced RAMP-1 promoter activity to 7-fold above background levels (Fig. 3). Similar results were obtained when RAMP-1 promoter/lucifersase constructs were transfected into RAMP-1 positive mouse NIH 3T3 cells.

To determine if the RAMP-1 promoter demonstrates tissue specificity in transfected cell lines, we compared the transcriptional activity of the RAMP-1 promoter in transfected C2C12 myoblasts and a cell line known to not express RAMP-1 mRNA (HEK293). Luciferase analysis demonstrated that constructs containing 4.7 kb to 782 bp of RAMP-1 5' flanking region were transcriptionally inactive in HEK293 cells. A construct containing 470 bp of RAMP-1 5' flanking region demonstrated modest transcriptional activity while a construct containing 343 bp of 5' flanking region demonstrated significant promoter activity. The results of these experiments suggest that the RAMP-1 promoter region between -782 and -343 contains a regulatory element that represses RAMP-1 promoter activity in a non-RAMP-1 expressing cell line.

### Silencer activity of the RAMP-1 repressor element

**Figure 4:**
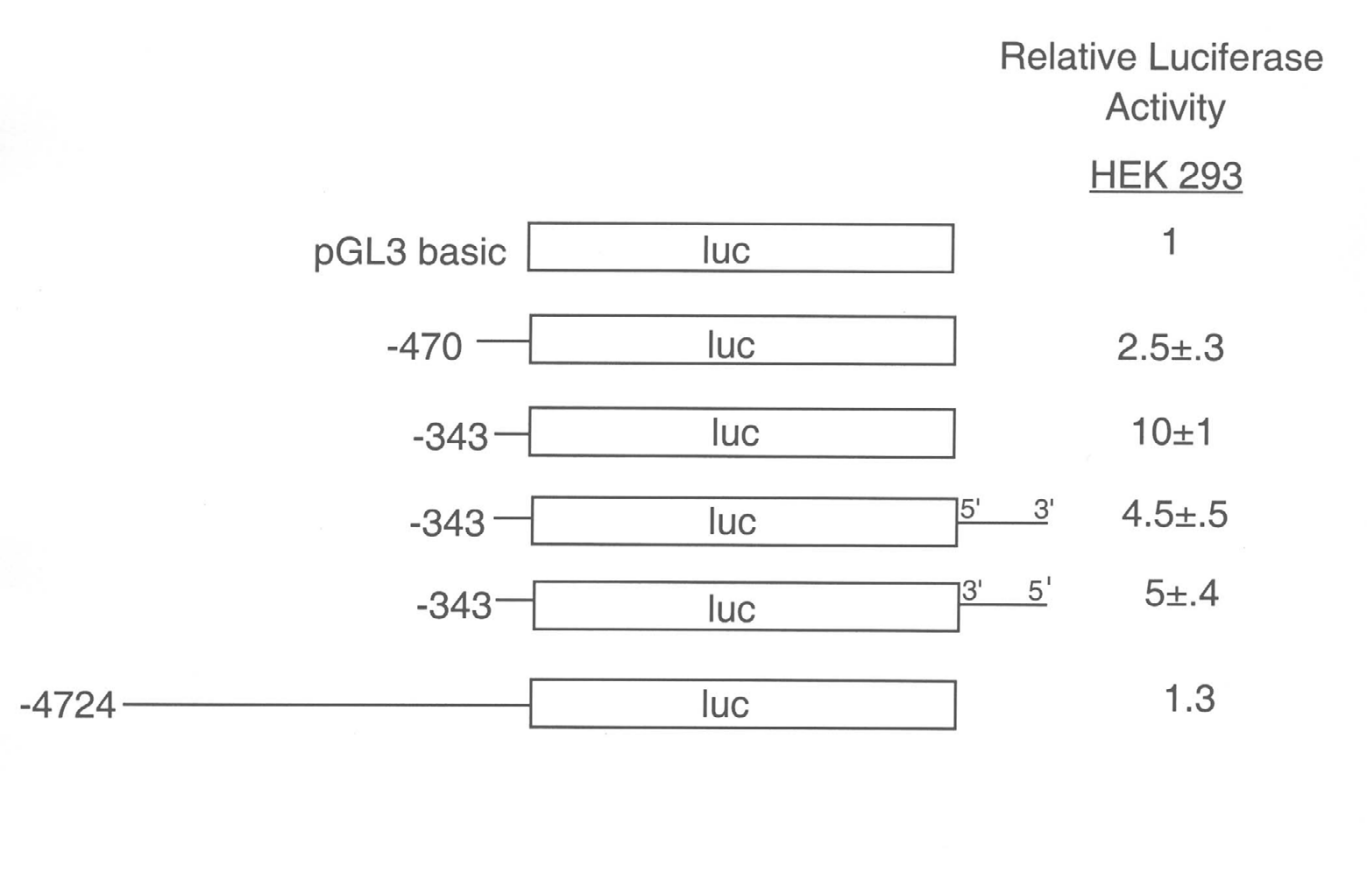
Silencer activity of RAMP-1 repressor element in transfected HEK293 cells. A RAMP-1 repressor element fragment (-783 to -343) was generated by PCR and ligated directly 3' to the luciferase gene in the RAMP-1 expression plasmid -343 RAMP-1/luciferase. The fragment was oriented in two different positions (5' to 3' and 3' to 5'). Relative luciferase activity was compared between these constructs, pGL3-basic, a plasmid containing 470 and 4.7 kb of RAMP-1 5' flanking region. Three independent sets of transfections were performed each giving similar results

We wished to determine if the RAMP-1 repressor element could repress RAMP-1 promoter activity in a position and orientation independent manner (silencer function). We therefore employed PCR to generate a fragment (-343 to -782 of the RAMP-1 promoter) containing the RAMP-1 repressor element. The fragment was then cloned in two orientations 3' to the luciferase gene in the -343 RAMP-1 promoter/luciferase construct. The expression of these constructs was compared to activity from the -343, -470 and -4724 RAMP-1 promoter/luciferase constructs in transfected HEK293 cells. Luciferase assays demonstrated that the repressor element reduced expression from the -343 RAMP-1/luciferase construct by approximately 50% (see Figure 4). An unpaired Student's t-test revealed that these differences were statistically significant (p < .002 and p < .004 for 5'-3' and 3'-5' silencer constructs, respectively). However, these constructs demonstrated significantly higher levels of activity than the -470 RAMP-1/luciferase construct (p < .006 and p < .003 for 5'-3' and 3'-5' silencer constructs, respectively). These results suggest that the RAMP-1 repressor element located between -343 and -782 bp of RAMP-1 5' flanking region has modest but statistically significant silencer activity and functions best in its native position.

## Discussion

Despite the important role RAMP-1 plays in CGRP cell signalling little is known about the transcriptional regulation of RAMP-1 gene. To date, no RAMP-1 gene promoter has been identified/cloned and RAMP-1 transcriptional initiation sites have not been determined in any RAMP-1 positive tissues. In the present study, we cloned the mouse RAMP-1 gene promoter and mapped multiple RAMP-1 transcriptional initiation sites in a variety of RAMP-1 positive tissues. Our data demonstrates that RAMP-1 transcriptional initiation occurs at similar sites and all within a 50 bp region in different RAMP-1 mRNA positive tissues.

To initiate studies on the transcriptional regulation of the mouse RAMP-1 gene during muscle differentiation, we examined RAMP-1 gene expression in C2C12 mouse myoblasts by semi-quantitative RT-PCR. Our results suggest that RAMP-1 gene expression is present in non-confluent, confluent and fused C2C12 cells. However RAMP-1 mRNA expression appears to be increased in confluent and fused C2C12 cells. Chakravarty et al. [23] demonstrated that a strong correlation exists between the level of RAMP-1 mRNA expression and CGRP binding. This suggests that the transcriptional activity of the RAMP-1 gene may control the responsiveness of tissues to CGRP. It will be important to determine if increased RAMP-1 mRNA expression in confluent and fused C2C12 cells results in significantly higher levels of CGRP receptor expression and increases in intracellular cAMP after treatment with CGRP. The role increased RAMP-1 mRNA gene expression plays in the process of C2C12 induced muscle differentiation is not yet clear. Nobel et al. [24] showed that CGRP causes increased creatine kinase activity in myoblast cultures. Creatine kinase activity is a well established marker of fused myoblasts (myotubes). It is reasonable to hypothesise that increased RAMP-1 gene expression in confluent C2C12s makes these cells more responsive to CGRP and thus may facilitate myoblast fusion.

Deletional mapping of the RAMP-1 gene promoter revealed that a 4.7 kb promoter fragment generates the highest levels of RAMP-1 promoter activity. However, a RAMP-1 promoter construct with only 343 bp of 5' region contains nearly 40% percent activity of the entire promoter. The delineation of such a small functional RAMP-1 promoter should facilitate the identification of DNA regulatory elements and the transcription factors they interact that activate RAMP-1 promoter activity in C2C12 cells.

Transfection of RAMP-1 promoter/luciferase constructs into RAMP-1 negative HEK293 cells demonstrated that a transcriptional control element present between -782 and -343 in the RAMP-1 promoter represses RAMP-1 promoter activity in the RAMP-1 negative-expressing cell line HEK293 cells. While this element appears to have modest silencing activity, our results suggest that full repressor activity of this element requires it to be in its native position within the RAMP-1 promoter. We hypothesise that negative-regulatory transcription factors, present in non-RAMP-1 expressing cells, bind to this element and repress RAMP-1 gene expression. Computer analysis of the 480 bp RAMP-1 repressor element has revealed potential binding sites for a variety of positive acting transcription factors such as MyoD, c-Myb, NF1 and Elk-1. Importantly, binding sites for two transcriptional repressors have also been identified: mammalian transcriptional repressor RBPJkappa/CBF (RBPJK) and bZIP domain, transcriptional repressor (E4BP4). Studies on the roles these transcription factors play in the repression of RAMP-1 gene expression are now underway.

## Conclusion

Promoter deletional analysis demonstrated that both positive and negative acting transcription factors regulate the tissue specificity of the RAMP-1 gene. DNase 1 protection and gel-shift experiments with extracts from C2C12 and HEK293 cells are underway to identify specific DNA sequences and the potential transcription factors they bind that mediate the activity of the RAMP-1 gene in RAMP-1 expressing and non- expressing cells. Once specific sequences/factors binding to the RAMP-1 repressor element are isolated, the effects that mutating these sequences has on RAMP-1 promoter activity in transgenic mice will be tested. These experiments will enable us to test the role such sequences/factors play in regulating the tissue-specific expression and activity levels of the RAMP-1 gene *in vivo*. Such transcriptional mechanism(s) are likely to play an important role in modulating the responsiveness of tissues to CGRP and may prove useful targets for future drug development aimed at regulating CGRP receptor gene expression.

## Methods

### 5' RACE analysis

5' RACE on total RNA from mouse heart, skeletal muscle and brain was performed employing a First Choice RLM-RACE kit (Ambion) according to manufacturers instructions. Briefly, 10 μg of total RNA from each tissue was treated with Calf Intestine Alkaline Phosphatase (CIP) for one hour at 37°C followed by extraction with phenol:chloroform and ethanol precipitation. The RNA was re-suspended in 11 μl of H_2_0. 5 μl of this RNA was then treated with tobacco acid pyrophosphate (TAP) for one-hour at 37°C. A 5' RACE adapter (provided in kit) was ligated to 5 μl of the TAP treated RNA. 2 μl of ligated RNA was subjected to reverse transcription (RT). RT products (1 μl) were subjected to two rounds of nested PCR according to manufacturers instructions. The first round of PCR employed an outer primer binding to the 5' RACE adapter (5'GCTGATGGCGATGAATGAACACTG 3') and an outer RAMP-1 gene specific primer (5' CGGGACCCTGACTATGGGAC 3'). The second round of PCR employed an inner primer binding to the 5' RACE adapter (5'GCTGATGGCGATGAATGAACACTG 3') and an inner RAMP-1 gene specific primer (5' TCTTCATGGTCACTGCCTGC 3'). Following the second round of PCR, PCR products were purified on an agarose gel and ligated into the PCR cloning vector pGEM-T Easy (Promega). A total of 8–10 clones representing each tissue were purified and subjected to DNA sequencing.

### Generation of RAMP-1 promoter/luciferase expression constructs

To generate RAMP-1 promoter/luciferase deletion constructs, PCR utilising an antisense primer corresponding to sequences -26 to -43 (see Fig. 1) and sense primers corresponding to -145 to -164; -324 to -343; -470 to -450; -760 to -782; -935 to -948; -1220 to -1243; -1574 to -1593; -1827 to -1846; -2032 to -2093; -2342 to -2365; -2533 to -2511; -4102 to -4079 and -4724 to -4702 was carried out as described in Jagger et al. [25]. The PCR fragments were cloned into the vector pGEM-T Easy (Promega) and sequenced. The plasmids were then cut with Not 1 and the released RAMP-1 promoter fragment filled in with Klenow, purified and cloned into the luciferase expression vector pGL3 basic (Promega).

To generate a construct containing the RAMP-1 repressor element cloned at the 3' end of the luciferase gene, the RAMP-1 promoter/luciferase construct containing 343 bp of 5' flanking region was cut with Bam H1. A region between -343 and -782 of the mouse RAMP-1 promoter was generated by PCR as above and cloned into the Bam H1 cut -343 RAMP-1 promoter/luciferase construct in two orientations.

### Tissue culture and RAMP-1/luciferase transfections

C2C12, HEK293 and NIH 3T3 cells were maintained in DMEM supplemented with 10% foetal calf serum, 100 μg/ml penicillin and 100 U/ml of streptomycin. To generate fused C2C12 myotubes, cells were allowed to become confluent and growth media was replaced with media containing only 1% foetal calf serum. For transient transfections in all the above cell lines, cells were transfected with 1.0–1.5 μg of specific RAMP-1/luciferase construct, 0.2 μg of a co-transfection control plasmid pRLTK (Promega) and Fugene 6 (Roche) according to manufacturers' instructions. Luciferase assays were performed employing a Dual Luciferase Reporter Assay System (Promega) and a Turner Design TD-20/20 luminometer.

### Semi-quantitative RT-PCR on RAMP-1 mRNA in differentiating C2C12 myoblasts

Total RNA from non-confluent, confluent and fused C2C12 myoblasts was isolated as above and reverse transcriptase (RT) reactions performed as previously described [25]. PCR amplification employing mouse RAMP-1 sense (5' CTATGGGGAGCTCACTTACTGC 3') and antisense (5'GCAGTCTTCCTTGGAGTTCAGAAT 3') oligos; mouse actin sense (5'GTTAACCAACTGGGACGACATGG 3') and anti-sense (5'GATCTTGATCTTCATGGTGC 3') mouse actin primers was performed as previously described [26]. A total of 30, 35 and 40 cycles were employed. The PCR products were run on a 1% agarose gel and Southern blotted to nylon membranes. The blots were probed with an end-labelled ^32^P actin probe (5' CACACTGTGCCCATCTACGA3') or RAMP-1 probe (5'GCCCAATCCGGAAGTGGACA 3') followed by autoradiography and densitomitry.

## Abbreviations

CGRP, calcitonin gene related peptide; CTR, calcitonin receptor; RAMP, receptor activity modifying protein; GPCR, G protein coupled receptor; CL, calcitonin receptor like

## Authors' contributions

RM performed the 5' RACE analysis and semi-quantitative RT-PCR. All other experiments were conducted by MP.

## Figures and Tables

**Table 1:** RAMP-1 5' RACE analysis. Total RNA was extracted from mouse brain, skeletal muscle and heart. RNA was treated with alkaline phosphatase, tobacco acid pyrophosphatase and ligated to a small adapter.  Following reverse transcription, multiple rounds of PCR were performed and the PCR products cloned and sequenced.  Transcriptional initiation sites are indicated relative to the start site of translation (ATG = 0).

Mouse RAMP-1 transcriptional start sites			
Clone	Brain	Muscle	Heart

1	-41	-50	-41
2	-41	-50	-41
3	-41	-40	-41
4	-9	-40	-9
5	-9	-50	-35
6	-11	-35	-35
7	-50	-9	-9
8	-41	-11	-50
9	-50	-	-41
10	-35	-	-
